# Solubility and Permeability Enhancement of Oleanolic Acid by Solid Dispersion in Poloxamers and γ-CD

**DOI:** 10.3390/molecules27093042

**Published:** 2022-05-09

**Authors:** Chiara De Stefani, Jessika Lodovichi, Laura Albonetti, Maria Cristina Salvatici, José Carlos Quintela, Anna Rita Bilia, Maria Camilla Bergonzi

**Affiliations:** 1 Department of Chemistry, University of Florence, Via U Schiff 6, 50519 Sesto Fiorentino, Florence, Italy; chiara.destefani@stud.unifi.it (C.D.S.); jessika.lodovichi@stud.unifi.it (J.L.); laura.albonetti@stud.unifi.it (L.A.); ar.bilia@unifi.it (A.R.B.); 2 National Research Council (CNR), Institute of Chemistry of Organometallic Compounds (ICCOM)—Electron Microscopy Centre (Ce.M.E.), Via Madonna del Piano 10, 50019 Sesto Fiorentino, Florence, Italy; salvatici@ceme.fi.cnr.it; 3 NATAC BIOTECH, Electronica 7, Alcorcón, 28923 Madrid, Spain; jcquintela@natacgroup.com

**Keywords:** γ-CD, oleanolic acid, PAMPA assay, poloxamers, solid dispersions

## Abstract

Oleanolic acid (OA) is a pentacyclic triterpenoid widely found in the Oleaceae family, and it represents 3.5% of the dry weight of olive leaves. OA has many pharmacological activities, such as hepatoprotection, anti-inflammatory, anti-oxidant, anti-diabetic, anti-tumor, and anti-microbic activities. Its therapeutic application is limited by its poor water solubility, bioavailability, and permeability. In this study, solid dispersions (SDs) were developed to overcome these OA limitations. Solubility studies were conducted to evaluate different hydrophilic polymers, drug-to-polymer ratios, and preparation methods. Poloxamer 188, Poloxamer 407, and γ-CD exhibited the highest increases in terms of OA solubility, regardless of the method of preparation. Binary systems were characterized using differential scanning calorimetry (DSC), X-ray diffraction (XRPD), and Fourier transform infrared spectroscopy (FTIR). In addition, pure compounds and SDs were analyzed using scanning electron microscopy (SEM) in order to observe both the morphology and the particle surface. In vitro dissolution studies were performed for P407, P188, and γ-CD SDs. Preparation using the solvent evaporation method (SEM) produced the highest increase in the dissolution profiles of all three polymers with respect to the OA solution. Finally, the effect of SDs on OA permeability was evaluated with an in vitro parallel artificial membrane permeability assay (PAMPA). The formulation improved passive permeation across the simulated barrier due to OA increased solubility. The dissolution and PAMPA results indicate that the amorphization of OA by SD preparation could be a useful method to enhance its oral absorption, and it is also applicable on an industrial scale.

## 1. Introduction

Oleanolic acid (OA) is a pentacyclic triterpenoid isolated from several food and medicinal plants [1]. It is widely distributed in plants of the Oleaceae family such as the olive plant [2,3]. It has been used as a remedy for liver diseases for over 20 years in China due to its hepatoprotective activity [4]. The potential therapeutic effect of OA on a wide range of diseases is attributable to its pharmacological activities, such as its anti-oxidant, anti-tumor, anti-inflammatory, anti-diabetic, and anti-microbial activities [5]. OA belongs to the Biopharmaceutics Classification System (BSC) class IV, and it has a very low water solubility of about 1 μg/mL and poor permeability [6,7], which usually results in low oral bioavailability and limited use in clinical treatments. Many attempts have been made to overcome these drawbacks, such as the use of solid lipid nanoparticles [8], nanosuspensions [9,10,11], self-nanoemulsified formulations [12], phospholipid complexes [13], and solid dispersions [14,15,16].

Currently, an efficient approach to gain enhanced dissolution and oral bioavailability of BCS II and IV class drugs is the use of amorphous solid dispersions [17,18].

Solid dispersion (SD) is defined as the dispersion of one hydrophobic active ingredient in an inert hydrophilic carrier in the solid state prepared using the melting method, solvent, or the melting solvent method. In these systems, the drug is combined with a water-soluble polymer to produce a single-phase amorphous mixture of the drug and the polymer [19]. The choice of carrier for the preparation of SDs is very important and directly affects the efficacy and stability of the formulation.

SDs have some advantages over other preparations used in the pharmaceutical technique field to improve the biopharmaceutical characteristics of poorly soluble compounds. In particular, it is a relatively simple preparation technique that produces the drug in an amorphous form, with a reduced particle size, improved wettability and dispersibility, and high porosity. All these aspects increase the solubility and dissolution profile of poorly soluble drugs, thereby improving the bioavailability of the drug. Furthermore, SDs can easily be formulated in solid pharmaceutical dosage forms, such as capsules and tablets [20], and they can be produced both in the laboratory and on industrial scales. Sildenafil [21,22], quercetin [23], ezetimibe [24], itraconazole [25], nisoldipine [26], diosmin [27], apigenin [28], diacerein [29], rosuvastatin calcium [30], and irinotecan [31] are some recent examples illustrating the improved in vitro and in vivo performance of drugs formulated in SD compared to the pure crystalline drug.

Povidone, polyethylene glycol, and calcium carbonate have been previously reported as successful applications for OA formulation. SDs increase its dissolution rate and oral bioavailability [13,14,32,33,34,35].

Some researchers have selected the PVPk30-Soluplus composite carrier to prepare OA SD, improving its dissolution rate [36]. A novel formulation of OA SD using leucine as the carrier to improve oral bioavailability has also been reported. Leucin produced an SD with good flowability, low hygroscopicity, and high bioavailability [15].

In the present study, we investigated hydrophilic polymers, which have not been tested for OA to date, to obtain SDs that increase the dissolution and permeability of this triterpene. The preparation of the formulation is part of a broader European project dealing with the valorization of olive leaf biomass. Each year, 4.5 million ton of olive leaves are produced in the world by the olive oil industry, and this biomass represents a problem for both the farmers and the whole olive oil industry. OA represents 3.5% of the dry weight of olive leaves (*Olea europea* L.) [37]. The preparation of amorphous SDs is currently one of the most useful tools in the pharmaceutical field to improve the drug bioavailability in both laboratorial and industrial scale processes. In this investigation, three drug-to-polymer weight ratios of 1:1, 1:2, and 2:1, and different SD preparation methods were considered. Differential scanning calorimetry, X-ray powder diffraction, Fourier transform infrared spectroscopy, scanning electron microscopy, and an in vitro dissolution study were employed for the characterization of the optimized formulations. The permeability of OA formulated as SD was tested using an in vitro parallel artificial membrane permeability assay and compared with that of OA solution.

## 2. Results and Discussion

### 2.1. Solubility Study of OA SDs

The hydrophilic polymers selected for SD preparation were Poloxamer 188 (P188), Poloxamer 407 (P407), Soluplus^®^, PEG 4000 (P4000), PEG 6000 (P6000), β-Cyclodextrin sulphated sodium salt (β-CDS), heptakis (2,3,6-tri-*O*-methyl)-β-Cyclodextrin (TM-β-CD), and γ-Cyclodextrin (γ-CD). The effects of the preparation method and drug-to-polymer ratio on OA solubility were investigated. Kneading (K) and the solvent evaporation method (SEM) [38] were used to prepare amorphous SDs and compared with the physical mixture (PM). A 1:1 drug-to-polymer weight ratio was initially considered. The results (Table 1, Table 2 and Table 3) indicate that the solubility of OA depends on both the polymer and the preparation method. All polymers except for β-CDS produce an increase in OA solubility, already highlighted in the case of PM.

P188, P407, TM-β-CD, and γ-CD show the highest solubility capacity in the case of the K method, and concerning the other polymers, the Poloxamers, PEG, and γ-CD show the highest solubility capacity using the SEM method. However, the polymers P188, P407, and γ-CD increase the OA solubility the most, regardless of the preparation method used. This effect becomes more evident with K and SEM, with values ranging from 42 to 74 μg/mL in the case of Poloxamers (K OA-P188, K OA-P407, SEM OA-P188, and SEM OA-P407), from 12 to 47 μg/mL with PEGs (K OA-P4000, K OA-P6000, SEM OA-P4000, and SEM OA-P6000), and from 44 to 110 μg/mL with γ-CD.

A drug-to-polymer 1:2 weight ratio was evaluated in the cases of P188, P407, P4000, P6000, and γ-CD (Table 2). The increased quantity of polymer results in enhanced OA solubility, particularly for both Poloxamers and in the case of SEM. OA solubility is 190 and 170 μg/mL for P188 and P407, respectively, and 145 μg/mL for γ-CD.

The 2:1 drug-to-polymer weight ratio does not improve the solubility of Poloxamer SDs, and the results are similar to those obtained with a 1:1 ratio (Table 3). Only in the case of γ-CD does the OA solubility reach about 220 μg/mL. P407 has a greater solubility capacity in all samples compared to P188, except in the case of SD with a drug-to-polymer weight ratio of 1:2 obtained with SEM. This could be related to the different HLB values of the two polymers, 22 for P407 and 29 for P188. P407 is less hydrophilic and more suitable for solubilizing highly lipophilic OA.

### 2.2. Characterization of OA SDs

The SDs that influenced the OA solubility the most were physically characterized. In particular, OA-P407 SDs, OA-P188 SDs, and OA-γ-CD SDs were considered.

#### 2.2.1. Differential Scanning Calorimetric Analysis (DSC)

**Figure 1:**
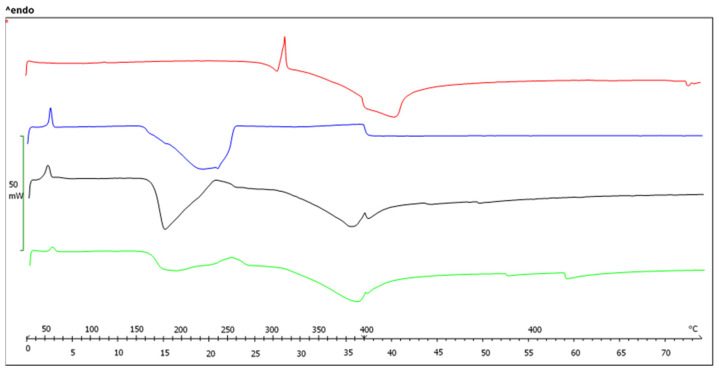
DSC thermograms of original OA (red), Poloxamer 407 (blue), PM OA-P407 1:2 (black), and SEM OA-P407 1:2 (green).

**Figure 2:**
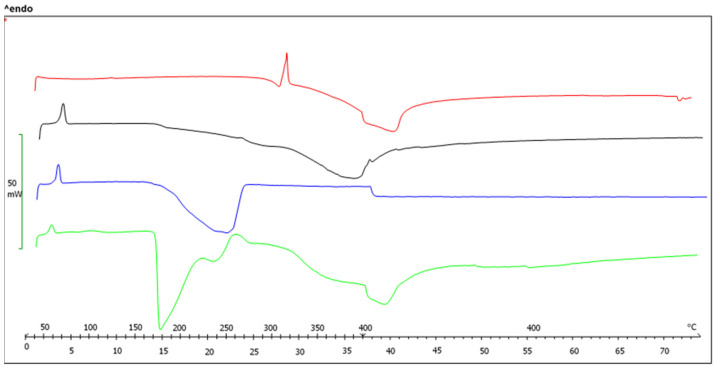
DSC thermograms of original OA (red), Poloxamer 188 (blue), PM OA-P188 1:2 (black), and SEM OA-P188 1:2 (green).

DSC analysis provides information about the amorphization of OA in the SDs. The DSC curves of OA, free carrier, OA-P407 SDs, and OA-P188 SDs are reported in Figure 1 and Figure 2. The pure OA exhibits an endothermic peak at 310 °C, corresponding to the melting point [8,34,38]. In the PM thermogram, the endothermic peak of OA disappears. This result may be due to interactions between OA with higher melting point and the polymer during the heating process [15]. The typical thermograms of SDs indicate the absence of the OA peak, which suggests that OA is completely soluble in the polymer phase and that it exists in an amorphous state in SDs.

#### 2.2.2. X-ray Diffraction (XRPD)

**Figure 3:**
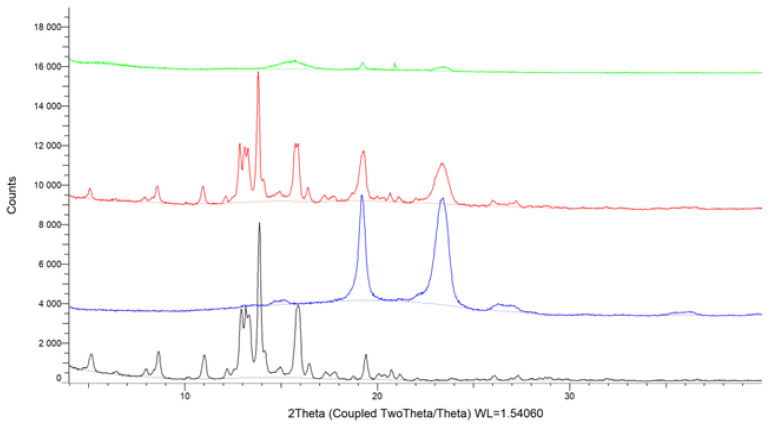
X-ray diffraction patterns of OA (black), Poloxamer 407 (blue), PM 1:1 (red), and SEM 1:1 (green).

XRPD was used to confirm the crystal form of free OA and its assumed structure in the SDs. The XRPD patterns of OA, free polymers, OA-P407 SDs, OA-P188 SDs, and γ-CD SDs are shown in Figure 3 and Figures S1 and S2 in the Supplementary Materials.

The pure OA exhibits strong sharp peaks at 5.14, 8.62, 11.02, 12.90, 13.13, 13.32, 13,85, 15.90, 16.45, and 19.43°, showing the crystalline state of the molecule. In the PMs, the diffraction peaks are less intense, but they can still be observed, indicating the presence of OA in the crystal form (Figure 3 and Figures S1 and S2 in the Supplementary Materials).

The diffractograms of OA SDs obtained with PM and K products are similar, with a peak shift in the range of 12–20°. This indicates the presence of an interaction between OA and the polymer, but no amorphization.

Amorphous materials present broad background signal patterns in the XRPD analysis, as evidenced in the pattern of the SEM products, where no distinct diffraction peaks are observed [34]. The X-ray diagrams of SDs show that the most intense peaks of pure OA decrease in intensity, pointing to a decrease in crystallinity depending on the preparation method used. The crystalline form of OA in SEM SDs changes, and OA is completely soluble in the polymer, which is consistent with the DSC data. These results indicate that OA is present in an amorphous form in the SDs, justifying the increased solubility and dissolution profile.

#### 2.2.3. Fourier Transform Infrared Spectroscopy (FTIR)

**Figure 4:**
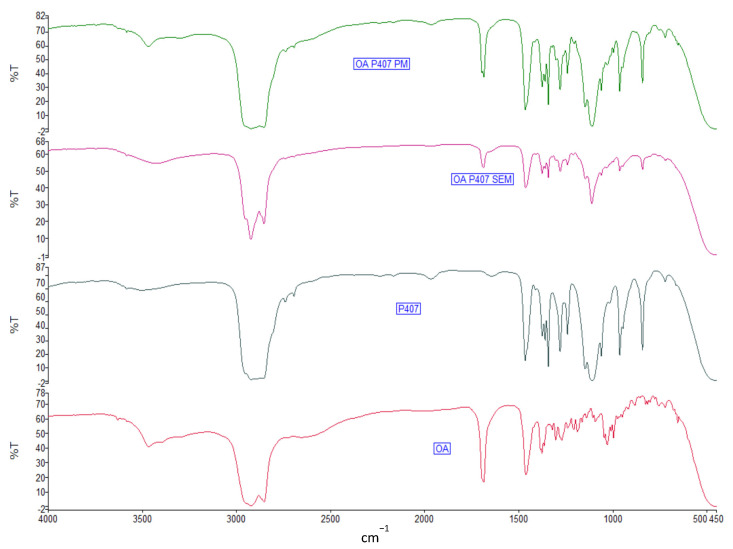
FTIR spectra of OA, P407, physical mixture (PM), and solid dispersion (SEM).

Figure 4 displays the FTIR spectra of original OA, P407, OA-P407 PM, and OA-P407 SEM, with a 1:2 drug-to-polymer weight ratio. The FTIR spectra of OA-P188 and OA-γ-CD SDs are reported in Figures S3 and S4 (Supplementary Materials). The spectrum of OA shows peaks of -OH stretching vibrations (3465 cm^−1^), C-H stretching vibrations (2944 cm^−1^), and -C=O stretching vibrations (1686 cm^−1^) [14]. P407 and P188 exhibit peaks of CH2 stretching vibrations (2921 cm^−1^), C-O stretching vibrations (1110 cm^−1^), and C-O-C linkage (963 cm^−1^) [39].

The FTIR spectra of PMs appear to be a sum of the OA and polymer spectra. In binary SD, the peaks of the -OH and -C=O stretching vibrations of OA shift to 3428 cm^−1^ and 1685 cm^−1^, respectively, suggesting that there might be H-bonding interactions between the groups of OA (-OH and -COOH groups) and the C-O-C and -OH groups of the Poloxamer (Figure 4).

The same shifts are evident in the case of P188 SD. The peaks of the -OH and -C=O stretching vibrations of OA shift to 3444 cm^−1^ and 1688 cm^−1^, respectively (Supplementary Materials Figure S3).

In the γ-CD FTIR spectrum, the peak at 3272 cm^−1^ is attributed to the -OH stretching vibration; the 2922 cm^−1^ peak is attributed to the stretching vibrations of -CH and -CH2; and the peaks at 1025, 1078, and 1154 cm^−1^ characterize the stretching vibrations of C-O [40]. After SEM, the OA peak of the -OH stretching vibration shifts to 3350 cm^−1^, and the peak of -C=O shifts to 1689 cm^−1^, which is indicative of interactions between OA and γ-CD (Supplementary Materials Figure S4).

#### 2.2.4. Scanning Electron Microscopy (SEM)

**Figure 5:**
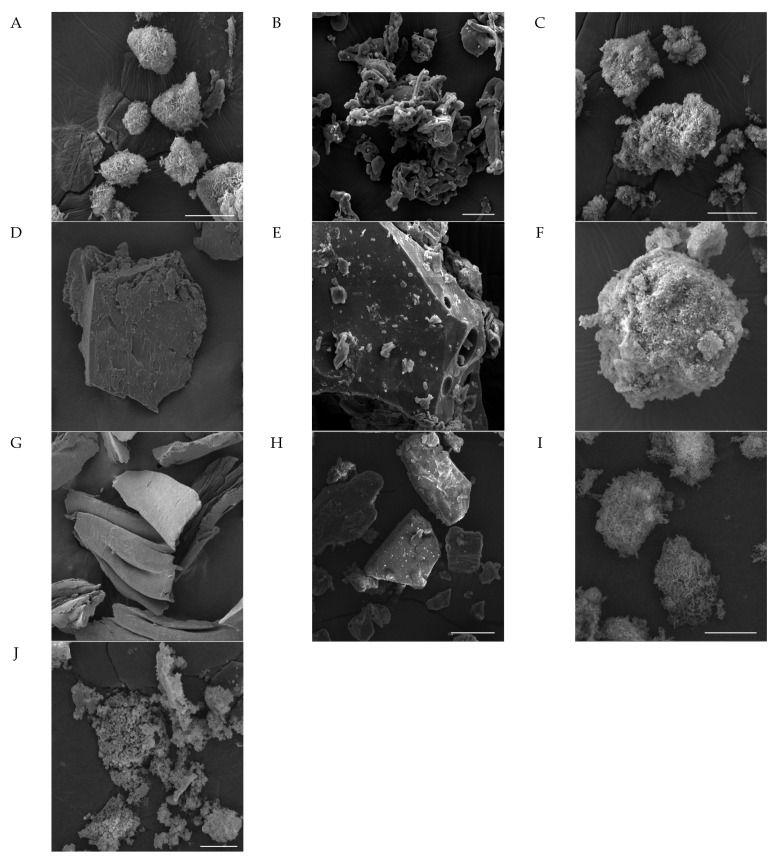
SEM micrograph of (**A**) OA, bar: 20 nm; (**B**) P407, bar: 20 nm; (**C**) PM OA-P407 1:2, bar: 20 nm; (**D**) SEM OA-P407 1:2, bar: 100 nm; (**E**) P188, bar: 20 nm; (**F**) PM OA-P188 1:2, bar: 20 nm; (**G**) SEM OA-P188 1:2, bar: 200 nm; (**H**) γ-CD, bar = 20 nm; (**I**) PM OA-γ-CD 1:2, bar: 10 nm; (**J**) SEM OA-γ-CD 1:2, bar: 10 nm.

The pure OA and OA SDs were analyzed using SEM in order to observe the morphology and the particle surface. The SEM images of OA-P407 1:1 SDs, OA-P188 1:1 SDs, and OA-γ-CD 1:1 SDs were analyzed and compared to those of pure OA and the polymers (Figure 5).

In terms of particle size, Poloxamer particles are larger than OA ones. The OA morphology shows that its particle size ranges from 10 to 100 µm and that the particle’s surface has a sponge structure with needle-like structures (Figure 5A). Poloxamer 407 has a spherical and smooth structure, with a particle size between 2 and 100 µm. These structures are elongated, filiform, and wrap around each other (Figure 5B). Furthermore, needle-like crystals and a sponge structure can be observed in the physical mixtures shown in Figure 5C,F,I. The sample PM OA-P407 is similar to the free OA; in fact, it has an amorphous rounded structure with a particle size ranging from 50 to 400 µm, and the surface of the particles has a needle-like structure (Figure 5C). SEM OA-P407 (1:1 *w*/*w*) has a smooth surface, and the particles have a flake structure with a smooth and non-porous surface. The particle size ranges from 100 µm to 1 mm (Figure 5D).

Poloxamer P188 has a smooth surface and a flake structure with a particle size ranging from 20 µm to 300 µm. Different from other polymers, it has a porous surface, and the hole size ranges from 5 µm to 20 µm (Figure 5E).

γ-CD has a crystalline structure, and PM OA-γ-CD has needle-like structures that aggregate into a large structure (Figure 5I). SEM OA-γ-CD shows an amorphous sponge-like structure without needle-like structures (Figure 5J).

The SEM analyses further demonstrate that OA is soluble in the carriers and in an amorphous state, with the sponge structures of OA being no longer observable. This is consistent with the results of the DSC and X-ray powder diffraction analyses.

#### 2.2.5. In Vitro Dissolution Testing

**Figure 6:**
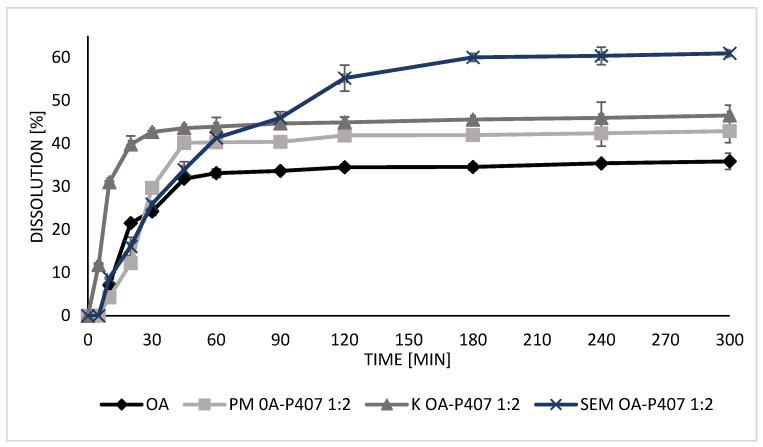
Dissolution profiles of OA, PM OA-P407 1:2, K OA-P407 1:2, and SEM OA-P407 1:2.

**Figure 7:**
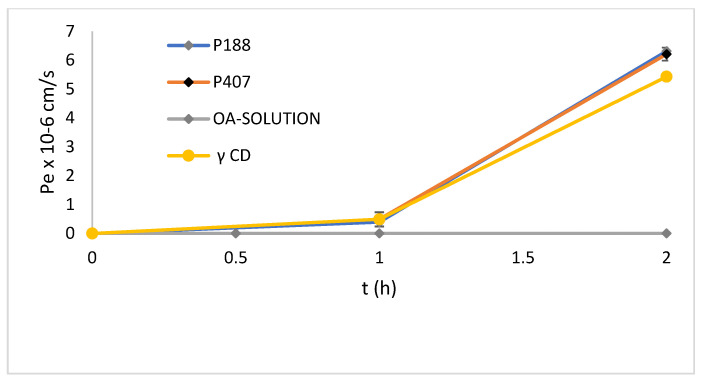
Effective permeability (Pe) of OA, SEM OA-P188 1:2, SEM OA-P407 1:2, and SEM OA-γ-CD1:2. (Data are expressed as mean ± SD of *n* = 3 experiments).

**Figure 8:**
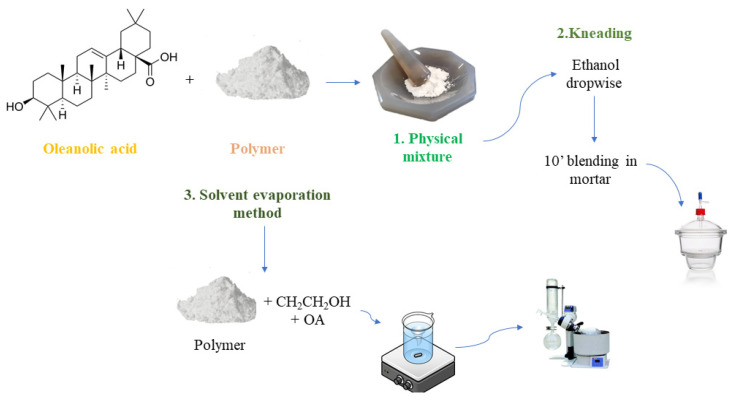
Schematic representation of preparation of the solid dispersions using physical mixture, kneading, and the solvent evaporation method.

The dissolution–time profiles of OA release from SDs were evaluated for P407, P188, and γ-CD polymers and compared with those of OA (Figure 6, Figure 7 and Figure 8). A dissolution medium containing 0.3% (*w*/*v*) sodium dodecyl sulfate (SDS) was used to provide sink conditions. Free OA is gradually released, and after 5 h, 35% dissolution is achieved.

More than 40% of OA is dissolved in PM OA-P407 in 5 h (Figure 6), indicating that the polymer already interacts in the PM with the OA and can improve the dissolution profile as previously observed in the solubility studies. Otherwise, OA release from K OA-P407 and SEM OA-P407 is fast, with maximum dissolution percentages of 46% and 61%, respectively, obtained in 5 h (Figure 6).

Moreover, P188 ameliorates the dissolution profile, obtaining 68% of the OA released in the case of SDs obtained with SEM. K and PM have the same effect, with about 50% of release at the end of the test (Supplementary Materials, Figure S5).

OA begins to release from γ-CD SDs after 30, 45, and 20 min for PM, K, and SEM respectively, followed by a gradual and prolonged release (Supplementary Materials, Figure S6). At the end of the test, the maximum dissolution percentage of the PM is 25%, compared to 48% for K and 49% for SEM.

For all three polymers, SEM provides a close drug-to-polymer interaction, which results in an improved OA dissolution profile with a higher percentage of OA in solution. This is because, in the SD system, OA uniformly dispersed in the polymer has improved wettability and dispersibility, and its crystalline state changes into a more soluble form, increasing its dissolution rate [15,17,18,34].

#### 2.2.6. In Vitro Parallel Artificial Membrane Permeability Assay (PAMPA)

PAMPA is a test for the rapid assessment of passive transport permeability. It permits a fast in vitro determination of the ability of molecules to permeate artificial membranes by passive diffusion, and, therefore, it gives useful information to estimate gastrointestinal absorption after oral administration [41]. The assay can be applied to single molecules, vegetable extracts, and formulations to evaluate the influence of the constituents of drug delivery systems on the permeability of the loaded substances [42,43,44,45,46]. In this study, PAMPA was used to evaluate the effect of SDs on OA passive permeability. The test evaluates the diffusion of OA from a donor compartment into an acceptor compartment. OA is a non-permeable molecule, as also proved by a Pe value of 2.7 ± 0.14 × 10^−7^ cm/s. The SDs improve the passive permeation of OA, with similar behavior of the two Poloxamers, and the final Pe value is 6.2 ± 0.22 × 10^−5^ cm/s and 6.3 ± 0.53 × 10^−5^ cm/s for P407 and P188, respectively, and 5.43 ± 0.12 × 10^−5^ cm/s in the case of γ-CD (Figure 7). A required recovery > 80% (84.5%) for an acceptable in vitro prediction is obtained [47]. The formulation improves the passive permeation across the simulated membrane barrier due to the increased solubility of OA.

## 3. Materials and Methods

### 3.1. Chemicals and Reagents

Oleanolic acid (OA, purity of 97%) was supplied by Natac Biotech SL (Alcorcón, Madrid, Spain). Poloxamer 188, Poloxamer 407, β-Cyclodextrin sulphated sodium salt, Heptakis (2,3,6-tri-*O*-methyl)-β-Cyclodextrin (TRIMEB), γ-Cyclodextrin, sodium dodecyl sulfate (SDS), and HPLC-grade solvents were obtained from Sigma Aldrich (Saint Louis, MO, USA) with the support of Sigma Aldrich Italia (Milan, Italy). Soluplus^®^ was provided by BASF (Ludwigshafen, Germany) with the support of BASF Italia, BTC Chemical Distribution Unit (Cesano Maderno, Monza e Brianza, Italy). PEG 4000 was purchased from Merck (Milano, Italy) and PEG 6000 from Galeno (Comeana, Prato, Italy). Distilled water was obtained from a Simplicity^®^UV Water Purification System, Merck Millipore (Darmstadt, Germany).

### 3.2. Preparation of Solid Dispersions

The preparation of the PM, K product, and SD obtained using SEM is reported in Figure 8. The PM was prepared by mixing ground OA and polymer in a mortar. The two components were mixed at drug-to-polymer weight ratios of 1:1, 1:2, and 2:1. For the preparation of the SD using K, PM was mixed with ethanol enough to maintain a slightly moist consistency. After 10 min of kneading in the mortar, the solid was placed in an airtight glass desiccator overnight. The SDs of OA polymers obtained using SEM were prepared by dissolving the polymer in ethanol and adding an appropriate amount of OA. The mixture was kept for 4 h at room temperature under magnetic stirring; then, ethanol was removed by rotary evaporation, and the solid was placed in an airtight glass desiccator overnight [15].

### 3.3. HPLC Analysis

An HPLC 1100 liquid chromatograph coupled with a DAD detector (Agilent Technologies, Palo Alto, CA, USA) was used for chromatographic analysis. The column was a Luna Omega Polar C-18 (150 mm × 4.6 mm, 3 µm) from Phenomenex (Castel Maggiore, Bologna, Italy) maintained at 25 °C. The wavelength of the detector was set at 210 nm. The mobile phase consisted of (A) formic acid/water pH 3.2 and (B) acetonitrile. The following isocratic method was applied: 0.10–25 min 20% A with a flow rate of 0.5 mL/min. The calibration curve was prepared using standard OA dissolved in methanol in a concentration range of 0.008–1 μg/μL, R^2^: 0.999.

### 3.4. Solubility Test

The solubility of pure OA and OA SDs was determined in distilled water. An excess amount of sample was dispersed in 2 mL of water and sonicated in an ultrasonic bath for 5 min. The dispersion was centrifugated for 15 min at 14,000 rpm and analyzed using HPLC-DAD analysis. All the solubility experiments were performed in triplicate.

### 3.5. Characterization of SDs

#### 3.5.1. Differential Scanning Calorimetric Analysis (DSC)

The thermograms of pure compounds and OA SD products were obtained using a Mettler TA4000 calorimeter equipped with a DSC25 cell (Mettler Toledo, Columbus, OH, USA). The samples, weighed with a Mettler M3 Microbalance (5–10 mg), were scanned in Al pans pierced with a perforated lid at 10 °C/min, from 30 to 400 °C, at a heating rate under static air.

#### 3.5.2. X-ray Powder Diffraction (XRPD)

The crystalline state of pure OA, hydrophilic polymer and OA SDs have been measured on a Bruker D8 Venture diffractometer at room temperature. Monochromatic Cu-Kα radiation (λ = 1.5406 Å) has been used in the 2θ angle range from 4° to 40° with a step width of 0.03°. The voltage and the current of the equipment were 40 mA and 40 kV.

#### 3.5.3. Fourier Transform Infrared Spectroscopy (FTIR)

Infrared spectra were obtained using an A L1600400 Spectrum two DTGS FTIR Perkin Elmer spectrometer (Perkin Elmer, Llantrisant, UK). A small quantity of single compounds or binary systems was mixed in a mortar with Nujol. The suspension was placed between two NaCl plates to obtain a homogeneous film. The scanning range was 4000 cm^−1^–400 cm^−1^, and data were collected with spectroscopy software Perkin Elmer Spectrum Version 10 (Perkin Elmer, Llantrisant, UK).

#### 3.5.4. Scanning Electron Microscopy (SEM)

The samples were analyzed using the Scanning Electron Microscope Gaia 3 (Tescan s.r.o, Brno, Czech Republic) Focused Ion Beam–Scanning Electron Microscope (FIB-SEM). The electron beam used for SEM imaging had a voltage of 10 kV and was operated in high vacuum mode with a secondary electron (SE) detector. Samples were deposited on a stub and coated with an ultrathin coating of silver. 

#### 3.5.5. In Vitro Dissolution Test

The in vitro dissolution of OA SDs was evaluated and compared with pure OA. The test was carried out according to European Pharmacopoeia and the study of Gao et al. using the paddle dissolver [34,48]. Type 1 gelatin capsules were filled with pure OA, with the OA ground in such a way as to have an amount of OA of 20 mg. The capsules were placed in 900 mL of an aqueous solution of SDS 0.3% *w*/*v* at 37 °C, and the paddle was set at 100 rpm. After 5, 10, 20, 30, 45, 60, 90, 120, 180, 240, and 300 min, 5 mL of dissolution medium was withdrawn, and 5 mL of fresh dissolution medium was added to each sample to maintain sink conditions. The samples were centrifuged for 5 min at 14,000 rpm and analyzed in HPLC. All experiments were performed in triplicate.

#### 3.5.6. PAMPA Assay

PAMPA was carried out on 96-well filter plates (Millipore, Billerica, MA, USA). An artificial membrane was derivatized with lecithin (1% *w*/*v*) and cholesterol (0.8% *w*/*v*) solution in 1,7-octadiene. Then, 10 μL of this solution was added to the PVDF membrane filters in the donor compartments. After the application of the artificial membrane, 0.25 mL of OA or SD solution in SDS 0.5% *w*/*v* was added to each well of the donor compartment, and 0.25 mL of 5% EtOH solution in PBS was added to each well acceptor compartment. Then, after placing the donor compartment into the acceptor compartment, the system was transferred into a sealed container and incubated at room temperature for 2 h. After the incubation, the OA concentrations in the donor and acceptor compartments were determined using HPLC. The effective permeability (Pe, cm/s) was calculated as previously reported [49,50]. Mass balance was also calculated. The assay was performed in triplicate.

## 4. Conclusions

In this study, SDs loaded with OA were successfully prepared. P188, P407, and γ-CD significantly improved the solubility of OA, particularly when using a 1:2 drug-to-polymer weight ratio and the solvent evaporation method. The optimized formulations were fully investigated and characterized using DSC, XRPD, FTIR, and SEM, which indicated that OA existed in an amorphous form and was dispersed homogenously in the polymer. The dissolution rate of OA was markedly enhanced by binary SDs with all three polymers. Furthermore, these improved the passive permeability of the molecules, as evidenced by the PAMPA experiments. The findings indicate that the amorphization of OA and its dispersion in the polymer using SD preparations could be a useful method to ameliorate the poor biopharmaceutical properties of a BCS class IV compound and a prerequisite for subsequent in vivo studies and clinical application.

## Figures and Tables

**Table 1:** Solubility of OA in hydrophilic polymers, using 1:1 drug-to-polymer weight ratio and different preparation methods (PM: physical mixture; K: kneading; SEM: solvent evaporation method).

Method	Polymer	Solubility (μg/mL)
**PM**	Poloxamer 188	17 ± 4
	Poloxamer 407	66 ± 6
	PEG 4000	12 ± 2
	PEG 6000	16 ± 2
	Soluplus	1.8 ± 0.3
	β-CDS	0.0 ± 0.0
	TM-β-CD	3.2 ± 0.6
	γ-CD	38 ± 3
**K**	Poloxamer 188	54 ± 4
	Poloxamer 407	47 ± 3
	PEG 4000	12 ± 1
	PEG 6000	16 ± 4
	Soluplus	2.7 ± 0.6
	β-CDS	3.9 ± 0.1
	TM-β-CD	31 ± 3
	γ-CD	44 ± 1
**SEM**	Poloxamer 188	42 ± 6
	Poloxamer 407	74 ± 2
	PEG 4000	30 ± 5
	PEG 6000	47 ± 1
	Soluplus	4.3 ± 0.4
	β-CDS	15 ± 1
	TM-β-CD	25 ± 2
	γ-CD	110 ± 0

**Table 2:** Solubility of OA in hydrophilic polymers, using 1:2 drug-to-polymer weight ratio and different preparation methods (PM: physical mixture; K: kneading; SEM: solvent evaporation method).

Method	Polymer	Solubility (μg/mL)
**PM**	Poloxamer 188	14 ± 2
	Poloxamer 407	32 ± 0.7
	PEG 4000	6 ± 0.8
	PEG 6000	5 ± 0.8
	γ-CD	66± 3
**K**	Poloxamer 188	79 ± 1
	Poloxamer 407	130 ± 7
	PEG 4000	12 ± 2
	PEG 6000	9 ± 2
	γ-CD	7 ± 0
**SEM**	Poloxamer 188	190 ± 42
	Poloxamer 407	170 ± 28
	PEG 4000	10 ± 1
	PEG 6000	12 ± 3
	γ-CD	145 ± 4

**Table 3:** Solubility of OA in hydrophilic polymers, using 2:1 drug-to-polymer weight ratio and different preparation methods (PM: physical mixture; K: kneading; SEM: solvent evaporation method).

Method	Polymer	Solubility (μg/mL)
**PM**	Poloxamer 188	13 ± 3
	Poloxamer 407	142 ± 5
	γ-CD	23 ± 1
**K**	Poloxamer 188	62 ± 8
	Poloxamer 407	188 ± 8
	γ-CD	34 ± 1
**SEM**	Poloxamer 188	36 ± 1
	Poloxamer 407	123 ± 6
	γ-CD	221 ± 17

## Data Availability

The data presented in this study are available on request to the corresponding author.

## Supplementary Materials

The following supporting information can be downloaded at: https://www.mdpi.com/article/10.3390/molecules27093042/s1, Figure S1. X-ray diffraction patterns of OA, Poloxamer 188, PM 1:2 and SEM 1:2; Figure S2. X-ray diffraction patterns of OA, γ-CD, PM 1:1 and SEM 1:1; Figure S3. FTIR spectra of OA, P188, physical mixture (PM), and solid dispersion (SEM); Figure S4. FTIR spectra of OA, γ-CD, physical mixture (PM), and solid dispersion (SEM); Figure S5. Dissolution profiles of OA, PM OA-P188 1:2, K OA-P188 1:2 and SEM OA-P188 1:2; Figure S6. Dissolution profiles of OA, PM OA-γ-CD 1:2, K OA-γ-CD 1:2 and SEM OA-γ-CD 1:2.

## Sample Availability

Samples of the OA SDs are available from the authors.
