# Supplementary material for: Solubility and Permeability Enhancement of Oleanolic Acid by Solid Dispersion in Poloxamers and γ-CD
Source: Molecules. 2022 May 9;27(9):3042. doi: 10.3390/molecules27093042 (PMC9101807; doi:10.3390/molecules27093042)
Supplement: Supplementary file 1 [file molecules-27-03042-s001.zip › molecules-1681615-supplementary.pdf]

molecules

Article

Solubility and Permeability Enhancement of Oleanolic Acid by Solid Dispersion in Poloxamers and γ-CD

Chiara De Stefani [1,†], Jessika Lodovichi [1,†], Laura Albonetti [1], Maria Cristina Salvatici [2], José Carlos Quintela [3], Anna Rita Bilia [1], Maria Camilla Bergonzi [1,*]

1 Department of Chemistry, University of Florence, via U Schiff 6, 50519 Sesto Fiorentino, Florence, Italy; chiara.destefani@stud.unifi.it (C.D.S.); jessika.lodovichi@stud.unifi.it (J.L.); laura.albonetti@stud.unifi.it (L.A.); ar.bilia@unifi.it (A.R.B.)
2 National Research Council (CNR), Institute of Chemistry of Organometallic Compounds (ICCOM)—Electron Microscopy Centre (Ce.M.E.), Via Madonna del Piano 10, 50019 Sesto Fiorentino, Florence, Italy; salvatici@ceme.fi.cnr.it
3 NATAC BIOTECH, Electronica 7, Alcorcón, 28923 Madrid, Spain; jcquintela@natacgroup.com
* Correspondence: mc.bergonzi@unifi.it; Tel.: +39-055-457-3678
† These authors contributed equally to this study.

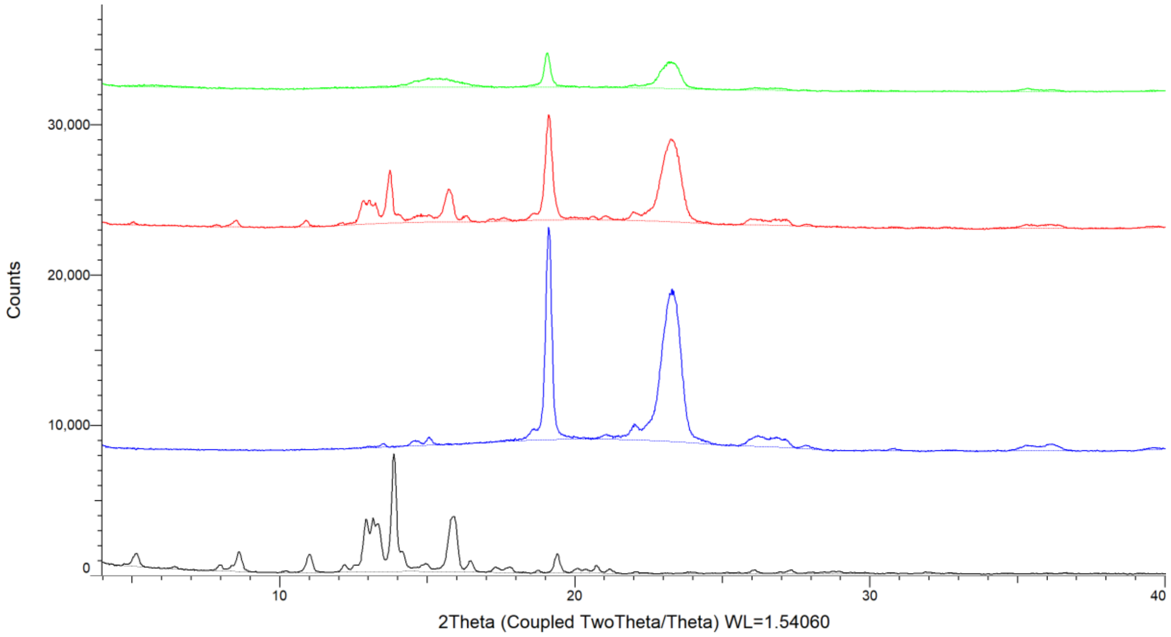

Figure S1. X-ray diffraction patterns of OA (black), Poloxamer 188 (blue), PM 1:2 (red) and SEM 1:2 (green).

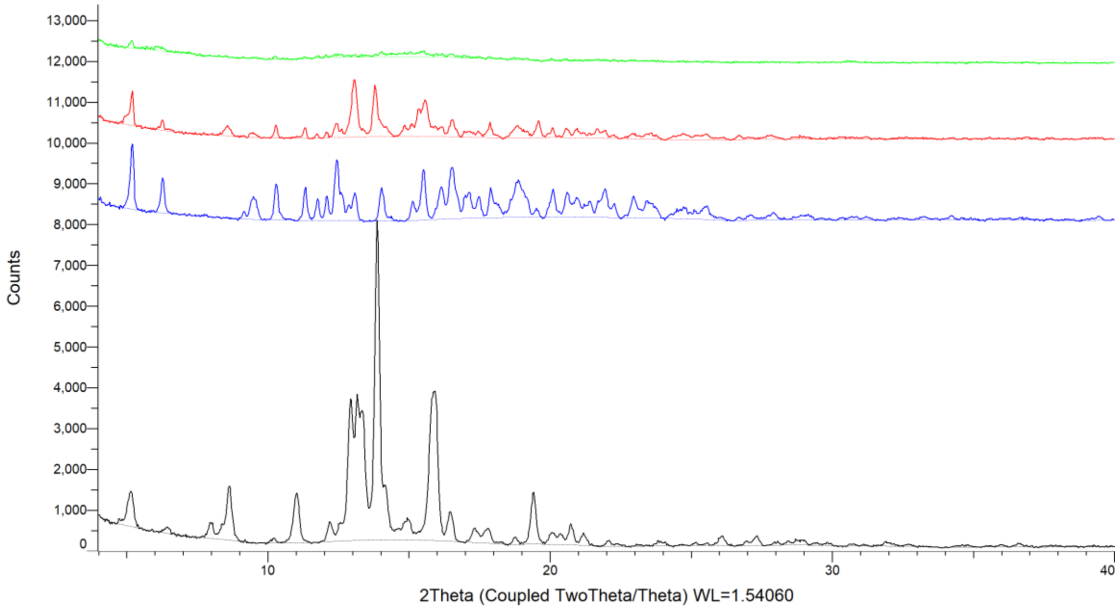

Figure S2. X-ray diffraction patterns of OA (black), γ-CD (blue), PM 1:1 (red) and SEM 1:1 (green).

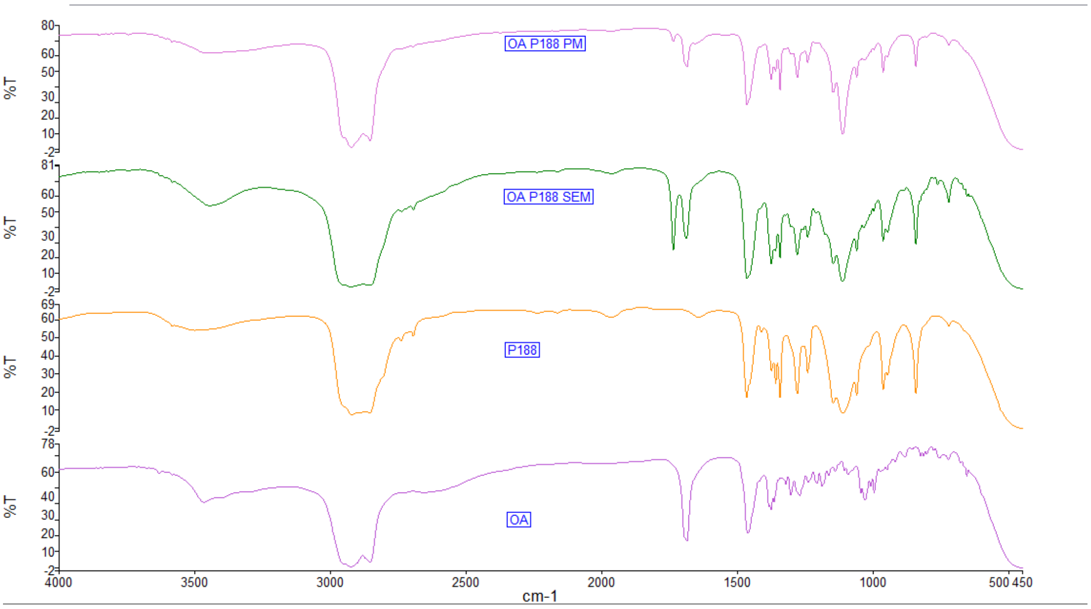

Figure S3. FTIR spectra of OA, P188 , physical mixture (PM), and solid dispersion (SEM).

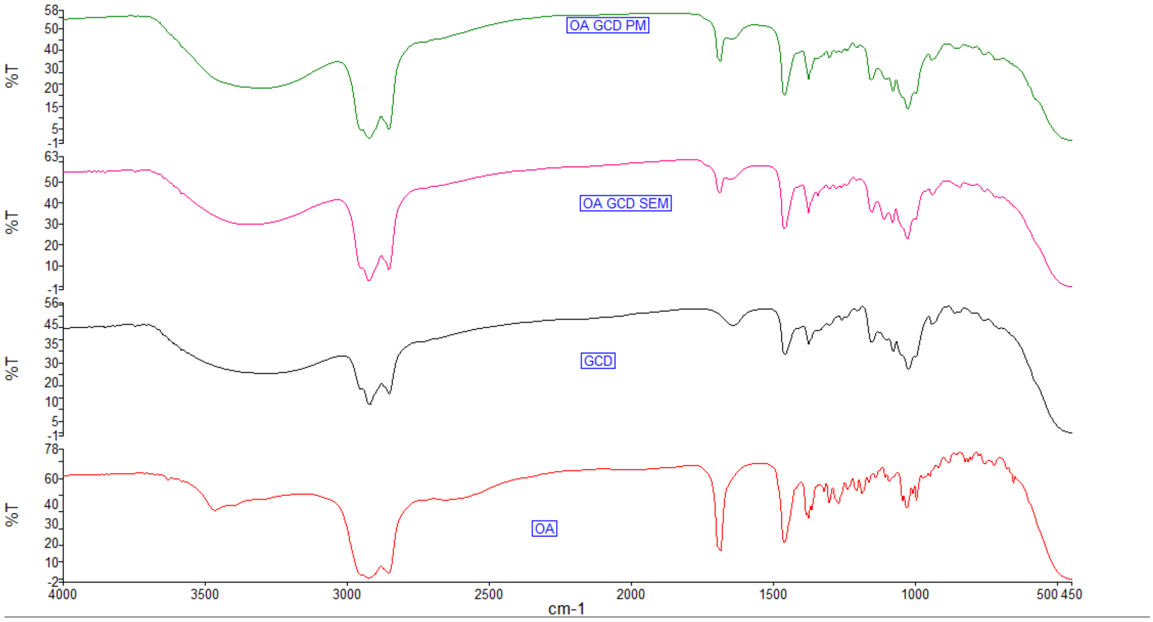

Figure S4. FTIR spectra of OA, γ-CD, physical mixture (PM), and solid dispersion (SEM).

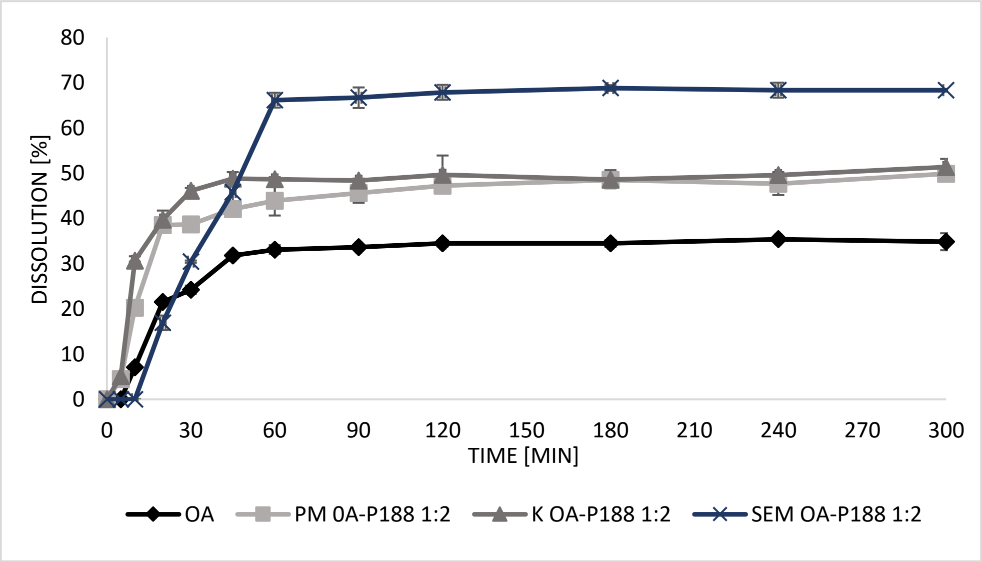

Figure S5. Dissolution profiles of OA, PM OA-P188 1:2, K OA-P188 1:2 and SEM OA-P188 1:2.

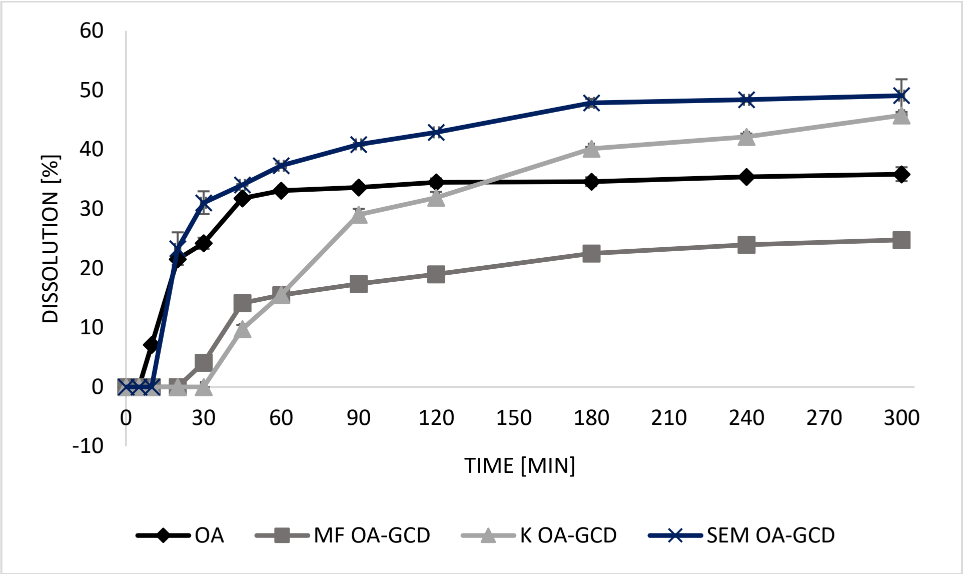

Figure S6. Dissolution profiles of OA, PM OA-γCD 1:2, K OA-γCD 1:2 and SEM OA-γCD 1:2.
